# Lower mental health related quality of life precedes dementia diagnosis: findings from the EPIC-Norfolk prospective population-based study

**DOI:** 10.1007/s10654-023-01064-7

**Published:** 2023-10-30

**Authors:** Renuka Chintapalli, Phyo K Myint, Carol Brayne, Shabina Hayat, Victoria L Keevil

**Affiliations:** 1 School of Clinical Medicine, University of Cambridge, Addenbrooke’s Hospital, Hills Road, Cambridge, England, UK; 2 Institute of Applied Health Sciences, University of Aberdeen, Aberdeen, Scotland, UK; 3 Cambridge Public Health, Department of Psychiatry, University of Cambridge, Herchel Smith Building, Forvie Site, Cambridge Biomedical Campus, Cambridge, CB2 0SZ England, UK; 4 Department of Behavioural Science and Health, Institute of Epidemiology and Health Care, University College London, London, England, UK; 5 Department of Medicine, University of Cambridge, Level 5 Addenbrooke’s Hospital, Hills Road, Cambridge, England, UK; 6 Medicine for the Elderly, Addenbrooke’s Hospital, Hills Road, Cambridge, England, UK

**Keywords:** Health-related quality of life, SF-36, Cognitive decline, Dementia, Mental health

## Abstract

Lower Health Related Quality of Life (HRQoL) precedes dementia in older adults in the USA. We explore prospective associations between HRQoL and dementia in British adults in mid and late-life, when interventions to optimise cognitive ageing may provide benefit. 7,452 community-dwelling participants (57% women; mean age 69.3 ± 8.3 years) attended the European Prospective Investigation of Cancer-Norfolk study’s third health check (3HC) and reported their HRQoL using Short-Form 36 (SF-36). Cox Proportional Hazard regression models explored associations between standard deviation differences in baseline Physical Component (PCS) and Mental Component Summary (MCS) scores, as well as eight SF-36 sub-scales (physical functioning, role-physical, bodily pain, general health, vitality, social functioning, role-emotional, mental health), and incident dementia over ten years. Logistic regression models explored cross-sectional relationships at the 3HC between HRQoL and objective global cognitive function (n = 4435; poor cognition = lowest performance decile). The cohort was examined as a whole and by age-group (50–69, ≥ 70), considering socio-demographics and co-morbidity. Higher MCS scores were associated with lower chance of incident dementia (Hazard Ratio [HR] = 0.74, 95% CI 0.68–0.81) and lower odds of poor cognition (Odds Ratio [OR] = 0.82, 0.76–0.89), with findings similar by age-group. Higher PCS scores were not associated with dementia in the whole cohort (HR = 0.93, 0.84–1.04) or considering age-groups; and were only associated with poor cognition in younger participants (OR = 0.81, 0.72–0.92). Similarly, associations between higher scores on subscales pertaining to mental, but not physical, HRQoL and lower dementia incidence were observed. Lower mental HRQoL precedes dementia diagnosis in middle-aged and older British adults.

**Supplementary Information:**

The online version contains supplementary material available at 10.1007/s10654-023-01064-7.

## Introduction

Globally, almost 10 million new dementia cases are diagnosed annually, and the estimated prevalence of dementia is projected to rise three-fold in the next 30 years [1]. Major financial costs, in the form of unpaid caregiving, are associated with dementia and the condition imparts a psychological, physical and social burden on individuals as well as wider society. Despite the increasing demand for curative therapies, none exist and there remains significant interest in strategies to delay or prevent cognitive decline in later life.

Dementia is often associated with a long prodromal period, with neurodegenerative changes and sub-clinical cognitive deficits starting decades prior to clinical manifestations [2]. Yet, screening for dementia is not recommended [3]. Dementia is a clinical syndrome rather than one particular entity, there are difficulties in discriminating the prodromal period from normal cognitive ageing, and there is insufficient evidence that earlier diagnosis leads to benefit [4]. Some studies have investigated interventions to delay and/or prevent dementia onset, but to date results from existing single and multidomain interventional studies have been inconsistent [5, 6]. This is despite observational evidence that building higher cognitive reserve [7, 8], engaging in socially and mentally stimulating activity [9, 10], having a wide social network, and increasing physical activity [11] are potentially modifiable risk factors; and considering that dementia prevalence today is lower than previously forecasted, perhaps due to advances in cardiovascular disease prevention [12, 13].

Evaluating interventions to slow, delay or prevent cognitive decline and dementia often necessitates large sample sizes and long follow-up periods. Interventional studies would benefit from easy-to-administer tools that can identify sub-groups within the general population who are most at risk of cognitive decline and, thus, most likely to benefit from potential preventative strategies. Physical and mental health impairments have been shown to precede dementia onset, for example instrumental activities of daily living disability [14] and lower objective cognitive performance [15]. However, there has been less focus on the utility of tools assessing functional health, such as health related quality of life (HRQoL), investigation of which might offer greater understanding and insights into dementia progression that could help inform future risk models. HRQoL evaluates physical and mental health impairments in the context of social functioning and considering the patient’s perspective [16]. HRQoL can be assessed using tools such as the 36 item Short Form Survey (SF-36). The SF-36 has been shown to be appropriate, valid and reliable for use in older populations with normal cognition or mild cognitive impairment. Previous work has also identified associations between SF-36 and incident diseases, such as stroke and myocardial infarction [17, 18], as well as IADL disability [19], all themselves risk factors for dementia. Therefore, it is perhaps not surprising that links between SF-36 scores and incident dementia were recently reported in adults ≥ 65 years old in the USA [16, 20, 21].

Considering this background and the administrative simplicity of the SF-36 (enabling it to be applied at scale and with the potential for remote administration), we extend this work to include a younger age cohort upon whom lifestyle interventions to delay/prevent dementia are most likely to be efficacious. We assess prospective associations between different domains of the SF-36, which capture different facets of mental and physical functional health, and all-cause dementia over 10 years of follow-up in community-dwelling adults enrolled in a British prospective cohort study. We investigate if relationships vary with age (50–69 versus ≥ 70 years) and explore the cross-sectional associations between SF-36 scores and cognitive function, measured objectively in a subset of in participants who completed a detailed health examination alongside the SF-36 assessment. We also examine if key findings persist when measurement of HRQoL earlier in the EPIC-Norfolk study timeline is considered.

## Methods

### Study population and data collection

**Fig. 1:**
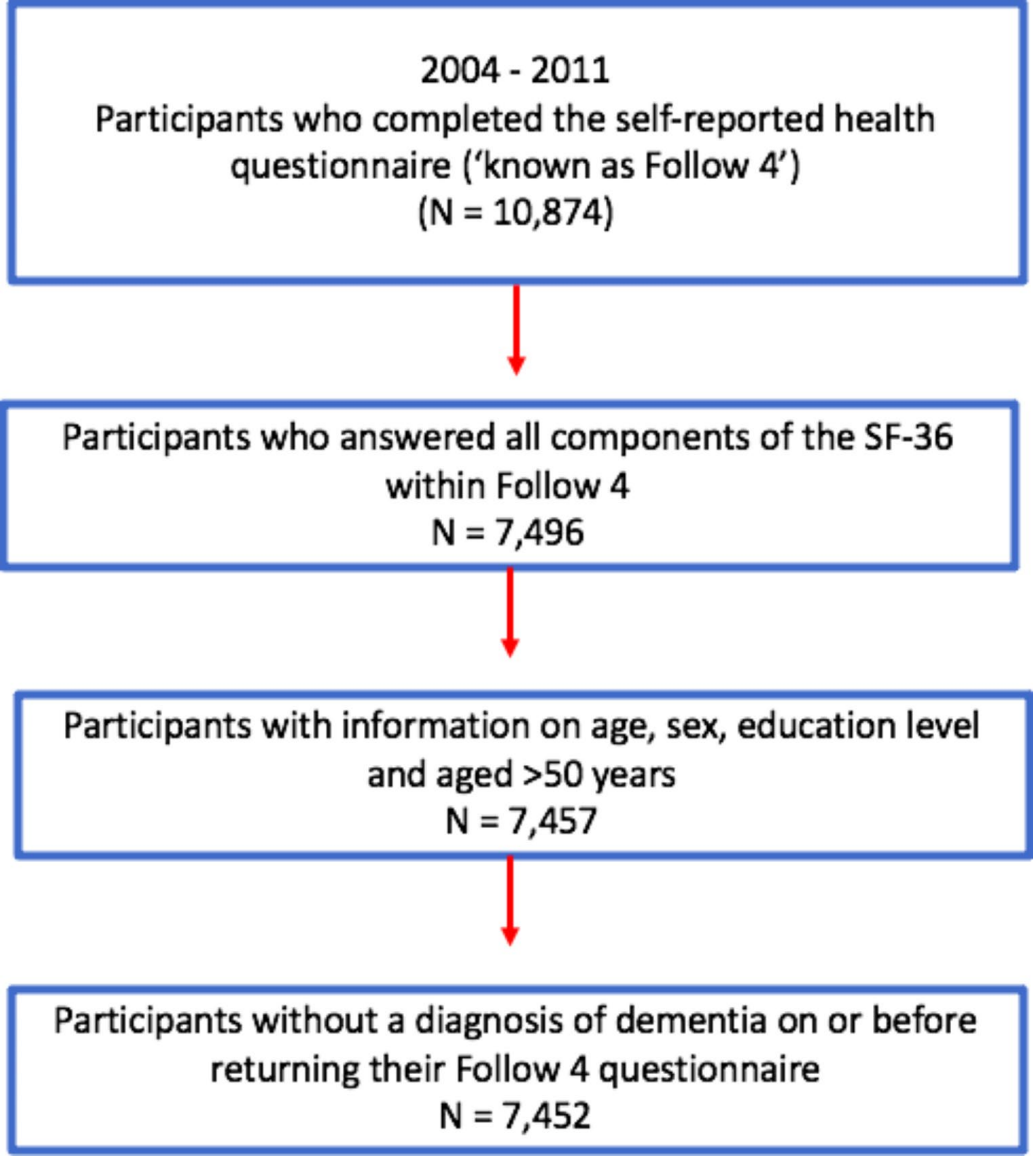
Selection of study participants in the EPIC-Norfolk third health check, 2006–2011 (including pilot phase 2004–2006) [15, 22]. Participants who had enrolled in the study at baseline were invited to complete a self-reported health and lifestyle questionnaire (known as ‘Follow 4’) around the same time as they were invited to attend the study’s third health check clinic. Participants who completed essential sections of the Follow 4 questionnaire, e.g., SF-36 components, were included in this study

The European Prospective Investigation of Cancer (EPIC) is a European-wide study of diet and health of which EPIC-Norfolk is a collaborating centre. In brief, EPIC-Norfolk enrolled over 25,000 community dwelling adults aged 40–70 years at baseline (1993–1997), who were registered with a participating GP in and around the city of Norwich, Norfolk (UK) [15]. This study included participants who completed a fourth follow-up health and lifestyle questionnaire (known as ‘Follow 4’) and attended a clinic during the study’s 3rd health check (3HC), conducted predominantly between 2006 and 2011 [15] (with a small pilot phase conducted between 2004 and 2006). We excluded participants aged < 50 years at the time of the fourth follow-up questionnaire or who had already been diagnosed with dementia (Fig. 1).

### Health-related quality of life assessment

Details concerning the construction of the SF-36 scales and summary scores, are outlined in other studies [23]. Briefly, the taxonomy comprises 3 levels: (1) 36 items, (2) 8 subscales scored from 0 to 100, calculated using 2–10 items each (physical functioning, role limitations due to physical problems [role physical], bodily pain, general health perception, vitality, social functioning, role limitations due to emotional problems [role emotional] and mental health), and (3) a physical component summary (PCS) and mental component summary (MCS), that aggregate sub-scales. The eight scales are hypothesized to form two distinct higher ordered clusters according to the physical and mental health variance that they have in common. PCS and MCS scores are then calculated based on USA-specific norms [16]. Higher scores in all subscales and summary components indicate better HRQoL in that domain.

The SF-36 was used to ascertain HRQoL as part of the EPIC-Norfolk study’s fourth follow-up health and lifestyle questionnaire, which coincided with the 3HC clinic.

### Dementia ascertainment

Incident dementia cases were defined as those participants free of dementia on the date they answered the Follow 4 questionnaire who were subsequently identified with a dementia diagnosis through capture of diagnoses in linked routine health records (determined using the ICD-10 codes listed in Supplementary Table S1). No distinction was made between dementia types in our analyses. Participants were followed up from the date of the fourth follow-up questionnaire until the first date of dementia diagnosis, date of death or censoring, or until 31 March 2019 if neither occurred. Definite clinical diagnosis with all causes of dementia was used in this study and the sub-types of dementia were not analysed separately.

### Cognitive function tests

The EPIC-Norfolk cognition battery consisted of 8 tests, measuring ability across different cognitive domains, assessed as part of the 3HC clinic appointment. These tests have been described previously [24] and are summarised in Supplementary Table S2. The following cognitive tests were included: short-form Extended Mental State Exam (SF-EMSE), the Hopkins Verbal Learning Test (HVLT), the Cambridge Neuropsychological Test Automated Battery Paired Associates Learning Test First Trial Memory Score (CANTAB-PAL FTMS), the PW Letter Cancellation Task, the Event and Time Based Task, the Visual Sensitivity Test (VST)-Simple, the Visual Sensitivity Test (VST)-Complex and a shortened version of the National Adult Reading Test (short-NART).

### Covariates

Co-variates were chosen a priori after reviewing confounders established in the literature [9, 25–29] and considering correlations between variables and potential overadjustment. The following variables were included: age, sex, body mass index (BMI), educational level (no formal qualifications versus at least completing school examinations aged 16 years), socioeconomic status, hearing problems, physical activity, smoking status, alcohol consumption, systolic blood pressure and self-reported comorbidity (cancer, myocardial infarction, stroke, bronchitis, asthma, arthritis, depression). Co-morbidity was represented as a dichotomous variable, as either having or not having any of the listed conditions. Ascertainment of covariate information is further detailed in the Supplementary Methods.

### Statistical analyses

All analyses were conducted using RStudio Version 4.1.0. Descriptive statistics were presented as means (standard deviation, SD), medians (interquartile range, IQR) or proportions (%; number, n) and compared using the relevant statistical test (t-test, Mann-Whitney U-test or chi-squared analysis). All association analyses were conducted as complete case analyses.

### HRQoL and its relationship to incident dementia

#### Main analyses

The prospective association between HRQoL and incident dementia was evaluated using Cox proportional hazards regression, with estimated hazard ratios (HR) and 95% confidence intervals (95% CI) reported. Time to event (follow-up time) for these models was defined as the time from the fourth follow-up questionnaire, to the date at which dementia was first diagnosed. Other participants were followed up until 31 March 2019 or to when the participant died or withdrew from the study. A priori models were adjusted for age and sex (Model 1); age, sex and education level (Model 2); age, sex, social class, smoking status and alcohol consumption (Model 3); age, sex, self-reported comorbidity and hearing problems (Model 4); and a final model including all of these covariates as well as waist-hip-ratio and systolic blood pressure (Model 5). Cox proportional hazards regression models assessed relationships between the two summary scores (PCS and MCS) and incident dementia, with multivariable adjustments as detailed above. Then relationships between the four subscales pertaining to physical health (physical functioning, role-physical functioning, bodily pain, general health) and incident dementia were assessed in separate models whilst additionally adjusting for MCS scores. Relationships between the four subscales pertaining to mental health (vitality, social functioning, role-emotional functioning, mental health) and incident dementia were similarly assessed in separate models whilst adjusting for PCS scores. This structure avoided model instability due to multiple moderate correlations between subscale scores. HRs estimating the relative instantaneous hazard (or reduction in the rate) of incident dementia for each standard deviation (SD) change in each subscale or summary score were calculated.

#### Additional Analyses

##### Sensitivity analysis: excluding dementia cases in the first five years of follow-up

Initial assessment of the Cox proportional hazards models suggested violation of the proportional hazards assumption with significant Schoenfeld residuals. Plots of scaled Schoenfeld residuals against time revealed stronger associations between MCS scores and dementia in the first five years of follow-up, although the best line of fit did not cross ‘0’ implying the direction of association remained unchanged.

Prospective analyses between HRQoL and incident dementia were repeated, after excluding participants diagnosed in the first five years of follow-up. For these models, Schoenfeld residuals were all non-significant (Model 5: Global test p = 0.41; MCS p = 0.14; PCS p = 0.52), with no evidence the PH assumption was violated. Hazard Ratios observed were computed and evaluated for consistency with the main analyses, i.e., those that included cases of dementia identified in the first five years of follow-up. These sensitivity analyses also address potential reverse causation bias, by excluding those diagnosed with dementia closest in time to assessment of HRQoL.

##### Deciles and quintiles of MCS and PCS

MCS and PCS scores were also treated in deciles and quintiles to plot the crude cumulative dementia incidence by decile or quintile of MCS and PCS scores. Following visualisation of these plots, the risk of incident dementia was further explored using Cox proportional hazards regression models with SF-36 summary scores in decile and quintile categories, rather than as a continuous variable.

##### HRQoL over the eleven years prior to follow-up for incident dementia

HRQoL measured during the Follow 4 questionnaire was the principal exposure measurement of our study. This coincided with the 3HC of EPIC-Norfolk, during which a significant proportion of the cohort underwent objective cognitive testing and were aged 48–92 years old. This wide age-range facilitated exploration of associations by age-group whilst considering adults < 50 years old are unlikely to experience dementia or be the focus of future preventative strategies or trials. However, given the longitudinal nature of the EPIC-Norfolk study, we additionally considered HRQoL measured during administration of a Health and Life Experiences Questionnaire 18 months after the first health examination (HLEQ1; 1993–1997; average time between HLEQ1 and Follow 4: 10.8 years, SD 1.9, range 4.5–15.6 years). Measurement of an exposure at repeated intervals increases measurement accuracy and allows consideration of change. HLEQ1 and Follow 4 scores for MCS and PCS were moderately correlated (Spearman’s rho MCS: 0.45, p < 0.001; PCS: 0.52, p < 0.001). We first explored the average MCS and PCS scores (sum of scores at each time point/2), representing average exposure over the eleven years preceding follow-up for incident dementia. HRs were calculated per SD change of average MCS and PCS score. We then explored change in HRQoL, as differences between HLEQ1 and Follow 4 Scores (‘stable’: +/- 5 point difference; ‘decrease’: >5 point lower score at Follow 4; ‘increase’: >5 point higher at Follow 4). HRs were calculated for ‘increase’ and ‘decrease’ categories relative to ‘stable’.

Model structures replicated those of the main analyses, with additional adjustment for MCS and PCS scores at HLEQ1, in the models considering change in HRQoL.

### HRQoL and its relationship to EPIC-Norfolk tests of cognitive function

Previous work in EPIC-Norfolk has already found strong associations between composite cognitive function and dementia [15], and served as a framework for our analytical approach. A composite cognitive function score was calculated for each participant by compiling scores from the aforementioned cognitive test battery and participants were then categorised into ‘good’ and ‘poor’ cognition groups as described in the Supplementary Methods. Briefly, ‘poor’ cognition was defined as being in the bottom decile for composite cognitive score, while ‘good’ cognition represents a score falling in all other deciles. The association between HRQoL and cognitive performance was studied using logistic regression correcting for the same covariates as above, reporting odds ratios (OR, 95% CI). ORs were calculated per SD change in each summary score of the SF-36 and represent the odds of poor cognitive performance.

## Results

### Demographic characteristics

The 7452 participants included had a mean age of 69.3 years (SD 8.3 years) and 57% were women. 511 participants had a record in their notes of incident dementia during an average of ten years of follow-up (maximum follow-up time 15.7 years; median 10.7 years). Table 1 describes the characteristics of participants at baseline and Supplementary Tables S3 and S4 further characterise the cohort by tertiles of MCS and PCS scores. Participants with higher PCS scores were notably younger, more likely to have formal qualifications and higher socioeconomic status, more likely to be never smokers, physically active, with lower BMI and waist-to-hip ratios and fewer co-morbidities, particularly arthritis. Variation in co-variables across tertiles of MCS scores was not as striking, but higher MCS scores were associated with being male, lower prevalence of depression, asthma and bronchitis, and a small but significant higher systolic blood pressure. Bivariate analyses comparing groups based on dementia status, showed that who developed dementia were more likely to be older, have no formal qualifications, have greater waist-to-hip ratios, perform manual labour, be physically inactive and non-drinkers and less likely to be current smokers and have higher systolic blood pressure readings at baseline. They were also more likely to have self-reported arthritis and hearing problems, and lower SF-36 summary and subscale scores (Supplementary Table S5). Similar cohort demographics for the cognitive assessment variables at the 3HC of EPIC-Norfolk have been published elsewhere [15].

**Table 1:** Demographic variables in the entire sample at the 3HC, at 31 March 2019

Variable	N (including = 7452)	Total (n = 7452)
**Age (years)**	7452	69.3 (8.3)
**Sex**	7452	
**Men**		3202 (43.0)
**Women**		4250 (57.0)
**BMI (kg/m** ^**2**^ **)**	6191	26.7 (4.2)
**Waist-hip ratio**	6185	0.89 (0.1)
**Education level**	7452	
No formal qualifications		1954 (26.2)
Formal qualifications		5498 (73.8)
**Socioeconomic status**	7385	
Non-manual		4917 (66.6)
Manual		2462 (33.4)
**Physical activity**	7451	
Inactive		2916 (39.1)
Moderately inactive		2103 (28.2)
Moderately active		1277 (17.1)
Active		1155 (15.5)
**Smoking status**	7442	
Current or former		3716 (49.9)
Never		3736 (50.1)
**Alcohol Units (median)**	7298	3.0 (8.0)
**Incident dementia cases**	7452	511 (6.9)
**Self-reported comorbidity**		
**Cancer**	7452	
Yes		616 (8.3)
**Myocardial infarction**	7452	
Yes		193 (2.6)
**Stroke**	7452	
Yes		97 (1.3)
**Bronchitis**	7452	
Yes		634 (8.5)
**Asthma**	7452	
Yes		695 (9.3)
**Arthritis**	7452	
Yes		2099 (28.2)
**Depression**	7452	
Yes		956 (12.8)
**Hearing problems**	7452	
Yes		2385 (32.0)
**Systolic blood pressure (mmHg)**	6201	136.0 (16.8)
**Median follow-up time (years)**	7452	10.0 (3.2)
**SF-36 summary scores**	7452	
PCS		47.0 (10.7)
MCS		54.3 (8.0)
**SF-36 subscale scores**		
Physical functioning	7452	75.1 (24.7)
Role-physical functioning	7452	72.0 (39.6)
Bodily pain	7452	76.4 (21.7)
General health	7452	72.0 (20.6)
Social functioning	7452	88.4 (20.3)
Role-emotional functioning	7452	88.0 (28.2)
Vitality	7452	62.7 (19.2)
Mental health	7452	79.2 (14.9)

Those who were missing comorbidity information were classed as not being diagnosed with the respective comorbidity. Values in brackets are SDs, interquartile range values or proportions, as appropriate. Abbreviations: BMI = body mass index, PCS = physical component summary, MCS = mental component summary.

### Prospective association between HRQoL and all-cause dementia

#### Main analyses

For the whole cohort, across all models, higher MCS score was associated with lower chance of dementia. In the final model, each SD increase in MCS score was associated with a 26% lower chance of dementia (HR = 0.74, 95% CI 0.68–0.81; p < 0.001). Findings were similar when the cohort was stratified into those aged 50–69 years and those aged ≥ 70 years (Table 2). There were no strong or consistent associations between PCS scores and incident dementia after adjustment for covariates. When stratified by age, there was a suggestion the relationship may differ between younger (50–69 years) and older (70 + years) cohort members but all associations remained non-significant.

**Table 2:** Association between SF-36 summary scores and incident all-cause dementia

SF-36 summary measure	Model 1			Model 2			Model 3			Model 4			Model 5		
	HR	95% CI	p	HR	95% CI	p	HR	95% CI	p	HR	95% CI	p	HR	95% CI	p
≥ 50 years															
N (n)		7452 (511)			7452			7221			7452			5995 (366)	
PCS	0.92	(0.84, 1.00)	0.05	0.92	(0.84, 1.00)	0.05	0.93	(0.85, 1.02)	0.12	0.89	(0.82, 0.98)	0.01	0.93	(0.84, 1.04)	0.21
MCS	0.75	(0.70,0.81)	< 0.001	0.75	(0.70, 0.81)	< 0.001	0.75	(0.70, 0.81)	< 0.001	0.75	(0.69, 0.81)	< 0.001	0.74	(0.68, 0.81)	< 0.001
50–69 years															
N (n)		4045 (77)												3447 (61)	
PCS	0.83	(0.68, 1.00)	0.06										0.89	(0.69, 1.16)	0.33
MCS	0.75	(0.63, 0.89)	0.001										0.75	(0.62, 0.92)	0.005
≥ 70 years															
N (n)		3407 (434)												2548 (305)	
PCS	0.93	(0.84, 1.02)	0.12										0.94	(0.83, 1.06)	0.32
MCS	0.75	(0.69, 0.82)	< 0.001										0.75	(0.68. 0.82)	< 0.001

Model 1: Age and sex. Model 2: Age, sex, education level. Model 3: Age, sex, socioeconomic status (manual vs. non-manual labour), smoking status (current/former vs. never), alcohol consumption (units). Model 4: Age, sex, self-reported comorbidity (cancer, myocardial infarction, stroke, bronchitis, asthma, arthritis, depression) and hearing problems. Model 5: Age, sex, socioeconomic status, smoking status, alcohol consumption, self-reported comorbidity, hearing problems, waist-hip ratio and systolic blood pressure. Each unit change in HR represents one SD change in the respective measure. N = Number of participants included in each respective model, n = number of incident cases of dementia. PCS = Physical component summary, MCS = Mental component summary

Higher scores on all mental health subscales were also individually associated with lower incident dementia, with particularly strong associations observed for role-emotional (HR = 0.78, 95% CI 0.72, 0.85), mental health (HR = 0.77, 95% CI 0.70, 0.85) and social functioning (HR = 0.77, 95% CI 0.69, 0.86) scores. There were no strong or consistent relationships with physical health subscales (Supplementary Table S6).

#### Additional analyses

The above analyses were repeated after excluding individuals who received a dementia diagnosis within five years of follow-up (Number of dementia cases = 409; 102 cases excluded). The direction of association for all analyses remained the same and strong associations were observed between higher MCS scores (HR 0.80 95%CI 0.82, 0.89) but not PCS scores (HR 0.92 95%CI 0.82, 1.04) and lower dementia. With respect to the SF-36 subscales, all results were consistent with the main analyses (Supplementary Tables S7 and S8).

Hazard function curves for cumulative incidence of dementia by MCS deciles and quintiles were explored. The relationship between higher MCS score and lower incident dementia appeared to be driven by the worst performers for mental HRQoL (see Supplementary Figures S1 & S3). Cox proportional hazards regression confirmed these visual interpretations. Supplementary Tables S9 and S10 show that compared to having scores in the mid-range of mental HRQoL (reference category: Deciles 2–9 or Quintile 3), those with scores in the lowest decile or quintile had highest incident dementia (Decile 1: HR = 2.12, 95% CI 1.57–2.86; Quintile 1: HR = 1.69, 95% CI 1.20–2.36). Those with scores in the highest decile or quintile had lower dementia incidence than the mid-range performers, suggesting variation across the whole range of MCS scores, but these associations were not as strong or consistent as those observed in the worst performing categories (Decile 10: HR = 0.65, 95% CI 0.46–0.93; Quintile 5: HR = 0.83, 95% CI 0.58–1.19).

Similar analyses for deciles or quintiles of PCS scores (Supplementary Figures S2 & S4) showed no associations after adjustment for age and sex (Table S9 and S10).

Table 3 presents associations between both average HRQoL and change in HRQoL, over 11 years, and incident dementia. MCS scores were 1.88 (SD 9.2) higher and PCS scores 2.55 (SD 9.4) lower at Follow 4 compared to HLEQ1. Consistent with main analyses, there were strong and consistent trends linking higher average MCS score and increasing MCS scores over time, with lower incident dementia; and decreasing MCS scores over time with higher chance of dementia. No strong or consistent trends were observed with PCS scores. Inferences were similar in age-stratified analyses. There was a possible strengthening of association between higher average PCS score and lower incident dementia amongst younger cohort members, but findings were not significant across all models (Supplementary Table S11). Analyses examining change in HRQoL category and incident dementia showed comparable trends in both age-groups, although categorical exposure analyses were limited by lower power in the younger age-group.

**Table 3:** Association between average and change in MCS and PCS scores, over eleven years, and incident all-cause dementia

Exposure	Model 1			Model 2**			Model 3**			Model 4**		Model 5**				
	HR	95% CI	p	HR	95% CI	p	HR	95% CI	p	HR	95% CI		p	HR	95% CI	p
N (n)		7212 (494)			7212 (494)			6993 (472)			7212 (494)				5815 (356)	
Average MCS*	0.80	(0.74, 0.87)	< 0.001	0.80	(0.74, 0.87)	< 0.001	0.80	(0.73, 0.87)	< 0.001	0.80	(0.73, 0.87)		< 0.001	0.79	(0.71, 0.87)	< 0.001
Average PCS*	0.92	(0.85, 1.01)	0.08	0.92	(0.85, 1.01)	0.08	0.94	(0.86, 1.03)	0.17	0.90	(0.82, 0.98)		0.02	0.95	(0.85, 1.06)	0.37
Change in MCS																
Increase	0.86	(0.68–1.08)	0.19	0.64	(0.48–0.84)	0.001	0.63	(0.47–0.83)	< 0.001	0.63	(0.48–0.83)		0.001	0.59	(0.43–0.82)	0.001
Stable (Ref)	1.00			1.00			1.00			1.00				1.00		
Decrease	1.47	(1.18–1.82)	< 0.001	1.45	(1.16–1.80)	< 0.001	1.40	(1.11–1.75)	0.004	1.45	(1.17–1.81)		< 0.001	1.50	(1.15–1.95)	0.003
Change in PCS																
Increase	1.15	(0.88–1.50)	0.31	1.05	(0.79–1.41)	0.73	0.99	(0.74–1.34)	0.97	1.04	(0.78–1.40)		0.77	0.97	(0.69–1.37)	0.86
Stable (Ref)	1.00			1.00			1.00			1.00				1.00		
Decrease	1.25	(1.03–1.52)	0.03	1.22	(1.00-1.50)	0.05	1.12	(0.91–1.37)	0.29	1.24	(1.02–1.52)		0.03	1.08	(0.85–1.37)	0.52

Model 1: Age and sex. Model 2: Age, sex, education level. Model 3: Age, sex, socioeconomic status (manual vs. non-manual labour), smoking status (current/former vs. never), alcohol consumption (units). Model 4: Age, sex, self-reported comorbidity (cancer, myocardial infarction, stroke, bronchitis, asthma, arthritis, depression) and hearing problems. Model 5: Age, sex, socioeconomic status, smoking status, alcohol consumption, self-reported comorbidity, hearing problems, waist-hip ratio and systolic blood pressure. N = Number of participants included in each respective model, n = number of incident cases of dementia. PCS = Physical component summary, MCS = Mental component summary. *Each unit change in HR represents one SD change in the respective measure; all co-variables measured at 3HC; **for change analyses: Models 2–5 are additionally adjusted for MCS/PCS scores at HLEQ1

### Cross-sectional association between HRQoL and global cognitive function

Table 4 outlines the cross-sectional association between SF-36 summary scores and global cognitive function at the 3HC. In fully adjusted models, each SD increase in MCS score was associated with lower odds of poor cognitive function (OR = 0.76, 95% CI 0.69–0.83), which persisted when the cohort was stratified by age-group. There was no association between PCS scores and global cognitive function in the whole cohort but higher PCS scores were associated with lower odds of poor cognition in those aged 50–69 years old.

**Table 4:** Association between SF-36 summary scores & composite cognitive score

SF36 summary measure	Model 1			Model 5		
	OR	95% CI	p	OR	95% CI	P
>=50 years						
** N**		4435			4293	
** PCS**	0.96	(0.89, 1.04)	0.35	0.93	(0.86, 1.02)	0.12
** MCS**	0.84	(0.78, 0.91)	< 0.001	0.82	(0.76, 0.89)	< 0.001
**50–69 years**						
** N**		2671			2593	
** PCS**	0.85	(0.76, 0.96)	0.007	0.81	(0.72, 0.92)	< 0.001
** MCS**	0.86	(0.72, 0.97)	0.01	0.83	(0.73, 0.93)	< 0.001
**>=70 years**						
** N**		1764			1700	
** PCS**	1.04	(0.94, 1.16)	0.45	1.02	(0.91, 1.14)	0.69
** MCS**	0.83	(0.75, 0.92)	< 0.001	0.83	(0.75, 0.92)	< 0.001

Model 1: Age and sex. Model 5: Age, sex, socioeconomic status, smoking status, alcohol consumption, self-reported comorbidity, hearing problems, waist-hip ratio and systolic blood pressure. ORs represent the odds of having a ‘poor’ composite cognitive score per SD change in the respective exposure measure. PCS = Physical component summary, MCS = Mental component summary

## Discussion

In a large, community-dwelling British cohort of adults aged ≥ 50 years, there is a strong association between higher mental HRQoL, derived from the SF-36, and lower chance of all-cause incident dementia. This relationship is as strong in those aged 50–69 years as in late-life (≥ 70 years). Consistently, we also report that higher scores in the subscales which contribute most to the MCS score are similarly associated with lower incident dementia, and associations persist in expected directions when average MCS score or change in MCS score over 11 years are considered. No strong relationships between PCS scores and incident dementia were observed. Interestingly, cross-sectional results for objective global cognitive function broadly mirror these findings. There are strong and consistent associations in the expected direction between mental HRQoL and cognitive function. However, higher physical HRQoL is only associated with lower odds of poor cognition in younger participants.

Our study has some limitations that should be considered when interpreting results. EPIC-Norfolk consists largely of white participants with smokers under-represented, limiting generalisability. We also did not study the association between HRQoL and the different subtypes of dementia, restricting aetiological insights. This may be especially relevant as the aetiology of dementia varies in younger and older cohorts, with younger adults more likely to have genetically-inherited forms of dementia [30]. Aetiological insights were also restricted as it was not possible to fully understand the causes of low HRQoL, although exploration of SF-36 subscale associations provided some insights in this regard. Inconsistencies in medical record keeping over time, due to changes in policy and practice within the NHS, could also lead to incomplete outcome ascertainment, and not all patients receive a formal dementia diagnosis. Despite this, our approach limited attrition bias [31] reflected in the range of cognitive abilities represented, and acquisition of dementia cases is likely to be highly specific [36]. We were also limited to the data collected. As with all observational studies, we are unable to account for all potential confounders, limiting exploration of all potential constructs underlying observed associations. We also only had objective measures of cognitive function in a subset of our cohort, and did not have multiple measures of cognition.

Despite these limitations, EPIC-Norfolk participants are part of a well-executed, detailed, longitudinal cohort study and our results are consistent with and extend findings from cohorts of older adults in the USA [16, 32]. Not only have we replicated results in a British cohort, but we report results from adults spanning a wide age-range followed up over an average of ten years. Thus, the present study provides evidence that lower mental HRQoL in mid-life, as well as in late-life, precedes dementia diagnosis by as much as ten years, in apparently ‘cognitively healthy’ community-based adults. These findings were also robust to assessment of average HRQoL, measured at two different time points in the EPIC-Norfolk study and consideration of change in HRQoL. Although HRQoL measured using the SF-36 at a single point in time has been established as an important potential health determinant [17], the higher chance of incident dementia with decreasing MCS score and lower chance with increasing MCS score was striking.

The strong and consistent relationships between mental HRQoL, and both incident dementia and poor cognition deserves consideration. Different factors are likely to influence HRQoL scores in mid and later life. For instance, younger adults may suffer more from employment-related issues [33], while older individuals are reported to experience higher rates of loneliness and isolation [34]. Our findings suggest the construct of interest may be the ability to respond to and mitigate these differing pressures, perhaps due to mental health conditions. Earlier occurrence of mental health issues in the timeline of pathological cognitive ageing and strong relationships between mental health and rate of cognitive decline have been previously reported on [35]. Participants in early stages of dementia might recognise, and therefore report, mental health problems over physical limitations. Similarly, more frequent social contact is associated with lower dementia risk and higher cognitive reserve [36]. Deterioration in mental health and social functioning may act as an ‘early warning sign’ of cognitive dysfunction, leading to a reduction in mental HRQoL that is evident before clinical signs of dementia.

In support of this, associations persisted after excluding participants diagnosed with dementia in the first five years of follow-up. Thus, it seems unlikely that our findings are explained solely by a high prevalence of undiagnosed, but potentially clinically detectable, dementia in our population. This is an important consideration since links between mental HRQoL and dementia may be bidirectional [36]. For example, links with social functioning could either be because social contact confers cognitive gain or because the capacity to maintain social contacts is a marker of cognitive resilience. Future work in cohorts with both baseline and follow-up measures of cognition and HRQoL could further explore the potential bidirectionality of associations.

It is also possible that both cognitive decline and mental health conditions have a shared aetiology. The mental health sub-scale, which significantly contributes to the MCS score, includes questions similar to those included in commonly used clinical anxiety and depression screening tools. Both the MH sub-scale and overall MCS scores have shown good sensitivity and specificity for detection of these conditions [37], which also have strong links with dementia and cognitive impairment [38]. Mental health conditions such as anxiety and depression deplete cognitive reserve, increasing risk for cognitive impairment in later life as well as exacerbating pre-existing age-related decrements in cognitive function. Although we adjusted for depression in our analyses, it is possible that the mental component part of the SF-36 was detecting subtle, pre-clinical signs of mental health deterioration. Stress, a determinant of HRQoL, can also lead to chronic inflammation, with ‘inflammaging’ implicated in both mental and cognitive health decline [39]. Further work perhaps investigating the different subtypes of dementia could further explore this but it was beyond the scope of this investigation.

With respect to physical HRQoL, we report a possible cross-sectional link between physical HRQoL and cognition in younger participants only, and no associations with dementia. This is broadly consistent with previous findings, although differences by age have not been previously explored [16, 20]. We can only speculate as to the reasons why this difference by age-group was observed. It may suggest that a greater proportion of the range of physical functional health in mid-life, compared to older age, relates to variation in cognitive function. For example, perhaps early cognitive decline is important in determining physical functional health at the high end of the normal range, which older adults are less able to attain due to age-related declines in physical functional health and/or higher prevalence of other age-related co-morbidities which influence physical HRQoL. Stronger associations at younger age might also be explained by the fact that physical impairment at those ages is rare and more likely to relate to incidence of specific diseases, rather than the age-related conditions and common co-morbidities adjusted for in our analyses. Thus, there may be a greater chance of residual confounding in younger compared to older participants. The lack of any consistent association between physical HRQoL and incident dementia is perhaps surprising given established links between physical and instrumental activities of daily living impairments and dementia [14, 40]. However, our findings with respect to incident dementia were robust to additional analyses, including HRQoL measured at two points in time.

Overall, our study has confirmed earlier reports of strong associations between higher mental HRQoL and lower incident dementia, extending this finding to a UK community-dwelling cohort in mid-late life [16, 32], over a median of ten years of follow-up. Although the authors are not recommending screening for dementia, these findings suggest the SF-36 may help identify populations most suitable for clinical trials of interventions to slow, delay or prevent dementia and optimise cognitive ageing. The relatively low respondent and administrative burden of the SF-36, in comparison to objective cognitive testing batteries, makes it an attractive alternative to assess the early indicators of future dementia onset [15], especially in the context of an ever-ageing population combined with increasing healthcare costs. However, we also recommend that other relatively low-burden, easily administered metrics of HRQoL (such as the EQ-5D [41] and EQ-VAS [42]) and patient reported outcomes are explored in this regard. In the absence of curative treatments for dementia, optimising cognitive health through provision of timely evidence-based interventions can offer large public health benefits, and enabling such trials to be conducted efficiently is critical to progress. Even if people never reach the diagnostic threshold for dementia, small deficits in cognitive function may have large effects on their ability to lead the best life possible.

Future investigation of links between HRQoL and dementia and cognitive function in other, diverse cohorts, including people of different ethnicities and cultural backgrounds, is required.

### Electronic supplementary material

Below is the link to the electronic supplementary material.

Supplementary Material 1
