# Supplementary material for: Lower mental health related quality of life precedes dementia diagnosis: findings from the EPIC-Norfolk prospective population-based study
Source: Eur J Epidemiol. 2023 Oct 30;39(1):67–79. doi: 10.1007/s10654-023-01064-7 (PMC10811145; doi:10.1007/s10654-023-01064-7)
Supplement: Supplementary file 1 — Supplementary Material 1 [file 10654_2023_1064_MOESM1_ESM.docx]

**Lower Mental Health Related Quality of Life Precedes Dementia Diagnosis: findings from the EPIC-Norfolk prospective population-based study.**

**Renuka D Chintapalli^1^, Phyo K Myint^2^, Carol Brayne^3^, Shabina Hayat^4^ and Victoria L Keevil^5,6^**

^1^ School of Clinical Medicine, University of Cambridge, Addenbrooke’s Hospital, Hills Road, Cambridge, England, UK

^2^ Institute of Applied Health Sciences, University of Aberdeen, Aberdeen, Scotland, UK

^3^ Cambridge Public Health, University of Cambridge, Strangeways Research Laboratory, Worts Causeway, Cambridge, England, UK

^4^ Department of Behavioural Science and Health, Institute of Epidemiology and Health Care, University College London, England, UK

^5^ Department of Medicine, University of Cambridge, Level 5 Addenbrooke’s Hospital, Hills Road, Cambridge, England, UK

^6^ Medicine for the Elderly, Addenbrooke’s Hospital, Hills Road, Cambridge, England, UK

Corresponding author: Dr Victoria L Keevil. Department of Medicine, University of Cambridge, Level 5 Addenbrooke’s Hospital, Hills Road, Cambridge, England, UK. Email: vlk20@cam.ac.uk

**Supplementary Methods**

**Calculating a composite cognitive score:**

For each of the individual cognition tests, a score of ‘0’ or ‘1’ was assigned based on whether the individual was in the ‘poor performance’ or ‘good performance or reference’ group for each of the eight cognitive outcome measures individually. The composite cognitive score was calculated as a sum of the score based on the performance group for all eight cognition test outcomes (range=0- 8). Participants were classified into two groups based on this score – those in the bottom decile (defined as having poor cognition) and those achieving a score above this cut-off (defined as having good cognition). The rationale for using percentile cut-offs in this population is that the prevalence of cognitive impairment using accepted standard diagnostic criteria is low and the cognitive scores were not normally distributed.

**Covariates:**

Education (the highest level attained) and social class were obtained from the baseline (1993- 1997) questionnaire. Education was categorized into three groups: 1) No qualification (not completing school up to the age of 16), 2) Completion of school up to the age of 16 or up to the age of 18; and finally 3) those obtaining an education to graduate level (those who obtained a degree or equivalent) or above. Social class was dichotomized, into ‘non manual’ and ‘manual’ class. The following variables were measured at the clinic appointment in the third phase of the study (3HC): Weight was measured to the nearest 0.1 kg (using digital scales, Tanita) and height was measured with a stadiometer (Chasmores, UK) to the nearest 0.1 cm to calculate body mass index (BMI: weight (in kilograms) divided by height (in meters squared)). Waist and hip circumference were measured to the nearest 0.1cm to calculate the waist-hip ratio. Physical activity levels were determined using the RUPE index^42,43^. Systolic blood pressure was also measured at this time. Self-report of smoking status (current, former or never smoker) and alcohol intake (Units/Week) were obtained from health and lifestyle questionnaire administered at the time of the clinic appointment. Medical history of heart-attack, stroke, cancer, diabetes, hypertension, chronic obstructive pulmonary disease (COPD), depression; and memory and hearing problems, were established using self-report of a range of conditions from health and lifestyle follow up questionnaire.

Education, social class and smoking were all treated as categorical variables in the analysis, as was co-morbidity (as present or not). BMI, waist-hip ratio, systolic blood pressure and alcohol consumption were entered as continuous variables. SF-36 scores were entered in terms of standard deviation changes, while the composite cognitive score was entered as a dichotomized variable based on the description above (poor performance or not).

**Missing data:** Missingness at the 3HC has previously been characterised by Hayat and colleagues^24,25^

**Supplementary Tables and Figures**

**Table S1.** International Classification of Disease (ICD) – 10^th^ revision.

| **ICD Code** | **ICD Description** |
| --- | --- |
| **F00** | Dementia in Alzheimer’s disease |
| **G30** | Alzheimer’s disease |
| **F00.0** | Dementia in Alzheimer’s disease with early onset |
| **G30.0** |  |
| **F00.1** | Dementia in Alzheimer’s disease with late onset |
| **G30.1** |  |
| **F00.2** | Dementia in Alzheimer’s disease, atypical or mixed type |
| **G30.8** | Other Alzheimer’s disease |
| **F00.9** | Dementia in Alzheimer’s disease, unspecified |
| **G30.9** |  |
| **F01** | Vascular dementia |
| **F01.0** | Vascular dementia of acute onset |
| **F01.1** | Multi-infarct dementia |
| **F01.2** | Subcortical vascular dementia |
| **F01.8** | Other vascular dementia |
| **F01.9** | Vascular dementia, unspecified |
| **F02** | Dementia in other diseases, classified elsewhere |
| **F02.0** | Dementia in Pick’s disease |
| **F02.1** | Dementia in Creutzfeldt-Jakob disease |
| **F02.2** | Dementia in Huntington’s disease |
| **F02.3** | Dementia in Parkinson’s disease |
| **F02.8** | Dementia in other specified diseases classified elsewhere |
| **G31.0** | Frontotemporal dementia |
| **G31.8** | Other specified degenerative diseases of nervous system. Grey-matter degeneration [Alpers]  Lewy body(ies)(dementia)(disease). Subacute necrotising encephalopathy [Leigh] |
| **F03** | Unspecified dementia |
| **F05.1** | Dementia superimposed on delirium |
| **F10.7** | Residual and late-onset psychotic disorder: Includes Alcoholic dementia NOS  Chronic alcoholic brain syndrome  Dementia and other milder forms of persisting impairment of cognitive functions |

**Table S2.** List of the individual cognitive tests used in the EPIC-Norfolk 3HC. Reproduced from Hayat et al, 2021^15^

| **Name of test** | **Predominant ability measured by test (Description of score)** |
| --- | --- |
| 1. A shortened version of the Extended Mental Status Exam (SF-EMSE) | Global function (continuous score) |
| 1. Hopkins Verbal Learning Test (HVLT) | Verbal episode memory (continuous score) |
| 1. Cambridge Neuropsychological Test Automated Battery Paired Associates Learning Test. First trial Memory Score (CANTAB-PAL FTMS) | Non-verbal episodic memory (continuous score) |
| 1. PW Letter Cancellation Task (PW-Accuracy Score) | Attention (continuous score) |
| 1. Event and Time Based Task (prospective memory) | Prospective memory (dichotomous outcome, success or fail) |
| 1. Visual Sensitivity Test (VST)-Simple | Simple and complex visual processing speed measured in milliseconds (continuous score) |
| 1. Visual Sensitivity Test (VST)-Complex |  |
| 1. Shortened version of the National Adult Reading Test (short-NART) | Reading ability and crystallised intelligence (continuous score) |

**Table S3.** Baseline characteristics by SF-36 MCS component score tertile

| **Variable** | **Total (n = 7452)** | **Tertile 1**  **0.0 – 53.8**  **(n=2552)** | **Tertile 2**  **53.9– 58.5**  **(n= 2482)** | **Tertile 3**  **58.6 – 74.0**  **(n=2424)** | **p-value** |
| --- | --- | --- | --- | --- | --- |
| **Age (years)** | 69.3 (8.3) | 68.6 (8.6) | 68.4 (8.0) | 70.9 (8.0) | <0.001 |
| **Sex** |  |  |  |  |  |
| Men | 3202 (43.0) | 997 (39.1) | 1083 (43.6) | 1124 (46.4) |  |
| Women | 4250 (57.0) | 1555 (60.9) | 1399 (56.4) | 1300 (53.6) | <0.001 |
| **BMI (kg/m^2^)** | 26.7 (4.2) | 26.4 (4.5) | 26.4 (4.0) | 27.0 (4.2) | <0.001 |
| **Waist-hip ratio** | 0.89 (0.1) | 0.89 (0.1) | 0.89 (0.1) | 0.90 (0.1) | <0.001 |
| **Education level** |  |  |  |  |  |
| No formal qualifications | 1954 (26.2) | 687 (26.9) | 601 (24.2) | 666 (27.5) |  |
| Formal qualifications | 5498 (73.8) | 1865 (73.1) | 1881 (75.8) | 1752 (72.2) | 0.02 |
| **Socioeconomic status** |  |  |  |  |  |
| Non-manual | 4917 (66.6) | 1685 (66.0) | 1651 (66.5) | 1581 (65.2) |  |
| Manual | 2462 (33.4) | 842 (33.0) | 806 (32.5) | 814 (33.6) | 0.66 |
| **Physical activity** |  |  |  |  |  |
| Inactive | 2916 (39.1) | 1037 (40.6) | 880 (35.5) | 1000 (41.3) |  |
| Moderately inactive | 2103 (28.2) | 688 (27.0) | 735 (29.6) | 680 (28.1) |  |
| Moderately active | 1277 (17.1) | 447 (17.5) | 459 (18.5) | 371 (15.3) |  |
| Active | 1155 (15.5) | 380 (14.9) | 407 (16.4) | 368 (15.2) | <0.001 |
| **Smoking status** |  |  |  |  |  |
| Current or former | 3716 (49.9) | 1313 (51.4) | 1224 (49.3) | 1179 (48.7) |  |
| Never | 3736 (50.1) | 1239 (48.6) | 1258 (50.7) | 1239 (51.1) | 0.13 |
| **Alcohol Units (median)** | 3.0 (8.0) | 2.0 (7.0) | 4.0 (9.0) | 3.0 (8.0) | <0.001 |
| **Self-reported comorbidity** |  |  |  |  |  |
| **Cancer** |  |  |  |  |  |
| Yes | 616 (8.3) | 219 (8.6) | 180 (7.3) | 217 (9.0) | 0.09 |
| **Myocardial infarction** |  |  |  |  |  |
| Yes | 193 (2.6) | 60 (2.4) | 60 (2.4) | 73 (3.0) | 0.42 |
| **Stroke** |  |  |  |  |  |
| Yes | 97 (1.3) | 37 (1.4) | 27 (1.1) | 33 (1.4) | 0.46 |
| **Bronchitis** |  |  |  |  |  |
| Yes | 634 (8.5) | 242 (9.5) | 201 (8.1) | 191 (8.1) | 0.03 |
| **Asthma** |  |  |  |  |  |
| Yes | 695 (9.3) | 271 (10.6) | 218 (8.8) | 206 (8.5) | 0.006 |
| **Arthritis** |  |  |  |  |  |
| Yes | 2099 (28.2) | 726 (28.4) | 618 (24.9) | 755 (31.1) | <0.001 |
| **Depression** |  |  |  |  |  |
| Yes | 1041 (14.0) | 588 (23.0) | 270 (10.9) | 183 (7.5) | <0.001 |
| **Hearing problems** |  |  |  |  |  |
| Yes | 2385 (32.0) | 839 (32.9) | 757 (30.5) | 789 (32.5) | 0.11 |
| **Systolic blood pressure (mmHg)** | 136.0 (16.8) | 135.1 (17.9) | 136.1 (16.6) | 137.2 (15.6) | <0.001 |
| **Mean follow-up time (years)** | 9.6 (2.7) | 6.8 (3.0) | 7.7 (2.6) | 8.0 (2.8) | <0.001 |
| **Incident Dementia** | 511 (6.9) | 213 (8.3) | 148 (6.0) | 156 (6.4) | 0.002 |

**Table S4.** Baseline characteristics by SF-36 PCS component score tertile

| **Variable** | **Total (n = 7452)** | **Tertile 1**  **0.0 – 45.3**  **(n=2460)** | **Tertile 2**  **45.4-53.7**  **(n=2535)** | **Tertile 3**  **53.8 -69.0**  **(n=2457)** | **p-value** |
| --- | --- | --- | --- | --- | --- |
| **Age (years)** | 69.3 (8.3) | 73.0 (8.3) | 68.8 (7.7) | 66.1 (7.3) | <0.001 |
| **Sex** |  |  |  |  |  |
| Men | 3202 (43.0) | 1004 (40.8) | 1114 (43.9) | 1084 (44.1) |  |
| Women | 4250 (57.0) | 1456 (59.2) | 1421 (56.1) | 1373 (55.9) | 0.03 |
| **BMI (kg/m^2^)** | 26.7 (4.2) | 28.0 (4.9) | 26.6 (4.0) | 25.5 (3.5) | <0.001 |
| **Waist-hip ratio** | 0.89 (0.1) | 0.91 (0.1) | 0.89 (0.1) | 0.88 (0.1) | <0.001 |
| **Education level** |  |  |  |  |  |
| No formal qualifications | 1954 (26.2) | 744 (30.2) | 668 (26.3) | 542 (22.1) |  |
| Formal qualifications | 5498 (73.8) | 1716 (69.8) | 1867 (73.6) | 1915 (77.9) | <0.001 |
| **Socioeconomic status** |  |  |  |  |  |
| Non-manual | 4917 (66.6) | 1579 (64.2) | 1660 (65.5) | 1678 (68.3) |  |
| Manual | 2462 (33.4) | 856 (34.8) | 853 (33.6) | 753 (30.6) | 0.007 |
| **Physical activity** |  |  |  |  |  |
| Inactive | 2916 (39.1) | 1347 (54.8) | 912 (36.0) | 656 (26.7) |  |
| Moderately inactive | 2103 (28.2) | 597 (24.3) | 737 (29.1) | 769 (31.3) |  |
| Moderately active | 1277 (17.1) | 308 (12.5) | 473 (18.7) | 496 (20.2) |  |
| Active | 1155 (15.5) | 208 (8.5) | 413 (16.3) | 534 (21.7) | <0.001 |
| **Smoking status** |  |  |  |  |  |
| Current or former | 3716 (49.9) | 1339 (54.4) | 1267 (50.0) | 1110 (45.2) |  |
| Never | 3736 (50.1) | 1121 (45.6) | 1268 (50.0) | 1347 (54.8) | <0.001 |
| **Alcohol Units (median)** | 3.0 (8.0) | 2.0 (7.0) | 3.0 (8.0) | 4.0 (9.0) | <0.001 |
| **Self-reported comorbidity** |  |  |  |  |  |
| **Cancer** |  |  |  |  |  |
| Yes | 616 (8.3) | 273 (11.1) | 190 (7.5) | 153 (6.2) | <0.001 |
| **Myocardial infarction** |  |  |  |  |  |
| Yes | 193 (2.6) | 124 (5.0) | 37 (1.5) | 32 (1.3) | <0.001 |
| **Stroke** |  |  |  |  |  |
| Yes | 97 (1.3) | 62 (2.5) | 20 (0.8) | 15 (0.6) | <0.001 |
| **Bronchitis** |  |  |  |  |  |
| Yes | 634 (8.5) | 297 (12.1) | 178 (7.0) | 159 (6.5) | <0.001 |
| **Asthma** |  |  |  |  |  |
| Yes | 695 (9.3) | 303 (12.3) | 224 (8.8) | 168 (6.8) | <0.001 |
| **Arthritis** |  |  |  |  |  |
| Yes | 2099 (28.2) | 1107 (45.0) | 672 (26.5) | 319 (13.0) | <0.001 |
| **Depression** |  |  |  |  |  |
| Yes | 1041 (14.0) | 405 (16.4) | 339 (13.4) | 297 (12.1) | <0.001 |
| **Hearing problems** |  |  |  |  |  |
| Yes | 2385 (32.0) | 957 (38.9) | 791 (31.2) | 636 (25.9) | <0.001 |
| **Systolic blood pressure (mmHg)** | 136.0 (16.8) | 136.6 (16.5) | 136.4 (16.0) | 135.5 (17.7) | 0.07 |
| **Mean follow-up time (years)** | 9.6 (2.7) | 8.6 (3.3) | 9.4 (2.8) | 9.8 (2.5) | <0.001 |
| **Incident Dementia** | 511 (6.9) | 257 (10.4) | 149 (5.9) | 105 (4.3) | <0.001 |

**Table S5.** Demographic variables in the entire sample at the 3HC, and comparison of participants based on dementia status at 31 March 2019.

| **Variable** | **N (including = 7452)** | **Total (n = 7452)** | **Incident dementia (n = 511)** | **No incident dementia (n = 6941)** | **p-value** |  |
| --- | --- | --- | --- | --- | --- | --- |
| **Age (years)** | | 7452 | 69.3 (8.3) | 76.5 (6.3) | 68.8 (8.2) | <0.001 |
| **Sex** | | 7452 |  |  |  |  |
| **Men** | |  | 3202 (43.0) | 222 (43.4) | 2980 (42.9) |  |
| **Women** | |  | 4250 (57.0) | 289 (56.6) | 3961 (57.1) | 0.91# |
| **BMI (kg/m^2^)** | | 6191 | 26.7 (4.2) | 26.4 (4.2) | 26.7 (4.2) | 0.99 |
| **Waist-hip ratio** | | 6185 | 0.89 (0.1) | 0.90 (0.1) | 0.89 (0.1) | 0.02 |
| **Education level** | | 7452 |  |  |  |  |
| No formal qualifications | |  | 1954 (26.2) | 168 (32.9) | 1786 (25.7) |  |
| Formal qualifications | |  | 5498 (73.8) | 343 (67.1) | 5155 (74.3) | <0.001^ |
| **Socioeconomic status** | | 7385 |  |  |  |  |
| Non-manual | |  | 4917 (66.6) | 342 (67.5) | 4575 (66.6) |  |
| Manual | |  | 2462 (33.4) | 165 (32.5) | 2297 (33.4) | <0.001# |
| **Physical activity** | | 7451 |  |  |  |  |
| Inactive | |  | 2916 (39.1) | 237 (46.4) | 2679 (38.6) |  |
| Moderately inactive | |  | 2103 (28.2) | 125 (24.5) | 1978 (28.5) |  |
| Moderately active | |  | 1277 (17.1) | 81 (15.9) | 1196 (17.2) |  |
| Active | |  | 1155 (15.5) | 68 (13.3) | 1087 (15.7) | <0.001^ |
| **Smoking status** | | 7442 |  |  |  |  |
| Current or former | |  | 3716 (49.9) | 282 (55.2) | 3434 (49.5) |  |
| Never | |  | 3736 (50.1) | 229 (44.8) | 3507 (50.5) | <0.001# |
| **Alcohol Units (median)** | | 7298 | 3.0 (8.0) | 2.0 (7.0) | 3.0 (8.0) | <0.001^ |
| **Self-reported comorbidity** | |  |  |  |  |  |
| **Cancer** | | 7452 |  |  |  |  |
| Yes | |  | 616 (8.3) | 43 (9.8) | 573 (9.5) | 0.20# |
| **Myocardial infarction** | | 7452 |  |  |  |  |
| Yes | |  | 193 (2.6) | 11 (2.5) | 182 3.0) | 0.68# |
| **Stroke** | | 7452 |  |  |  |  |
| Yes | |  | 97 (1.3) | 9 (2.0) | 88 (1.4) | 0.54# |
| **Bronchitis** | | 7452 |  |  |  |  |
| Yes | |  | 634 (8.5) | 43 (9.6) | 591 (9.6) | 0.96# |
| **Asthma** | | 7452 |  |  |  |  |
| Yes | |  | 695 (9.3) | 36 (8.1) | 659 (10.5) | 0.23# |
| **Arthritis** | | 7452 |  |  |  |  |
| Yes | |  | 2099 (28.2) | 176 (39.5) | 1923 (31.1) | <0.001# |
| **Depression** | | 7452 |  |  |  |  |
| Yes | |  | 956 (12.8) | 64 (14.1) | 892 (15.5) | 0.44# |
| **Hearing problems** | | 7452 |  |  |  |  |
| Yes | |  | 2385 (32.0) | 213 (42.9) | 2172 (32.4) | <0.001# |
| **Systolic blood pressure (mmHg)** | | 6201 | 136.0 (16.8) | 138.0 (17.6) | 136.0 (16.7) | 0.02 |
| **Mean follow-up time (years)** | | 7452 | 9.6 (2.7) | 7.4 (2.9) | 9.7 (2.6) | <0.001^ |
| **SF-36 summary scores** | | 7452 |  |  |  |  |
| PCS | |  | 47.0 (10.7) | 43.7 (11.0) | 47.3 (10.6) | <0.001 |
| MCS | |  | 54.3 (8.0) | 52.9 (9.1) | 54.4 (7.9) | <0.001 |
| **SF-36 subscale scores** | |  |  |  |  |  |
| Physical functioning | | 7452 | 75.1 (24.7) | 64.5 (27.3) | 76.0 (24.2) | <0.001 |
| Role-physical functioning | | 7452 | 72.0 (39.6) | 55.6 (43.7) | 73.2 (38.9) | <0.001 |
| Bodily pain | | 7452 | 76.4 (21.7) | 72.9 (22.8) | 76.7 (21.6) | <0.001 |
| General health | | 7452 | 72.0 (20.6) | 67.8 (21.1) | 72.3 (20.5) | <0.001 |
| Social functioning | | 7452 | 88.4 (20.3) | 82.2 (23.7) | 88.9 (20.0) | <0.001 |
| Role-emotional functioning | | 7452 | 88.0 (28.2) | 76.8 (37.7) | 88.9 (27.2) | <0.001 |
| Vitality | | 7452 | 62.7 (19.2) | 58.0 (19.7) | 63.1 (19.1) | <0.001 |
| Mental health | | 7452 | 79.2 (14.9) | 76.5 (16.0) | 79.4 (14.8) | <0.001 |

Those who were missing comorbidity information were classed as not being diagnosed with the respective comorbidity. Values in brackets are SDs, interquartile range values or proportions, as appropriate. #Chi-squared test. ^Mann-Whitney U-test. Abbreviations: BMI = body mass index, PCS = physical component summary, MCS = mental component summary.

**Table S6.** Association between SF-36 subscale measures and incident all-cause dementia.

|  |  | **Model 1** | | **Model 2** | | **Model 3** |  | **Model 4** |  | **Model 5** | |
| --- | --- | --- | --- | --- | --- | --- | --- | --- | --- | --- | --- |
| **SF-36 subscale** |  | **HR** | **95% CI** | **HR** | **95% CI** | **HR** | **95% CI** | **HR** | **95% CI** | **HR** | **95% CI** |
| **N (no. incident dementia cases)** | | 7452 (511) | | 7452 | |  | 7221 |  | 7452 | 5995 (366) | |
| **Physical functioning** | | 0.90 | (0.83, 0.98) | 0.90 | (0.83, 98) | 0.91 | (0.84, 1.00) | 0.89 | (0.82, 0.97) | 0.95 | (0.84, 1.06) |
|  | |  |  |  |  |  |  |  |  |  |  |
| **Role-physical functioning** | | 0.90 | (0.83, 0.98) | 0.90 | (0.83, 0.98) | 0.92 | (0.85, 1.01) | 0.90 | (0.82, 0.97) | 0.92 | (0.83, 1.01) |
|  | |  |  |  |  |  |  |  |  |  |  |
| **Bodily pain** | | 0.99 | (0.91, 1.08) | 0.99 | (0.91, 1.08) | 1.00 | (0.92, 1.00) | 0.99 | (0.90, 1.07) | 1.06 | (0.95, 1.18) |
| **General health** | | 0.93 | (0.85, 1.02) | 0.93 | (0.85, 1.02) | 0.94 | (0.85, 1.03) | 0.92 | (0.84, 1.01) | 0.93 | (0.82, 1.04) |
|  | |  |  |  |  |  |  |  |  |  |  |
| **Social functioning** | | 0.77 | (0.70, 0.83) | 0.76 | (0.70, 0.83) | 0.85 | (0.69, 0.82) | 0.76 | (0.70, 0.83) | 0.77 | (0.69, 0.86) |
|  | |  |  |  |  |  |  |  |  |  |  |
| **Role-emotional functioning** | | 0.78 | (0.73, 0.84) | 0.79 | (0.73, 0.84) | 0.79 | (0.74, 0.85) | 0.78 | (0.73, 0.84) | 0.78 | (0.72, 0.85) |
|  | |  |  |  |  |  |  |  |  |  |  |
| **Vitality** | | 0.85 | (0.76, 0.94) | 0.85 | (0.76, 0.94) | 0.82 | (0.68, 0.99) | 0.84 | (0.76, 0.94) | 0.84 | (0.74, 0.96) |
| **Mental health** | | 0.80 | (0.74, 0.87) | 0.80 | (0.74, 0.87) | 0.80 | (0.73, 0.86) | 0.80 | (0.73, 0.87) | 0.77 | (0.70, 0.85) |

Model 1: Age and sex. Model 2: Age, sex, education level. Model 3: Age, sex, socioeconomic status (manual vs non-manual labour), smoking status (current/former vs never), alcohol consumption (units). Model 4: Age, sex, self-reported comorbidity (cancer, myocardial infarction, stroke, bronchitis, asthma, arthritis, depression) and hearing problems. Model 5: Age, sex, socioeconomic status, smoking status, alcohol consumption, self-reported comorbidity, hearing problems, waist-hip ratio and systolic blood pressure. Each unit change in HR represents one SD change in the respective measure; physical health subscales additionally adjusted for Mental Component Summary score & mental health subscales for Physical Component Summary score.

**Table S7.** Sensitivity analysis for the association between SF-36 summary scores and incident

all-cause dementia (excluding those with dementia diagnosis in first five years after 3HC).

|  | **Model 1** | | | | **Model 5** | | | |
| --- | --- | --- | --- | --- | --- | --- | --- | --- |
| **SF-36 summary measure** | **N** | **HR** | **95% CI** | **p** | **N** | **HR** | **95% CI** | **P** |
| **PCS** | 7351 | 0.91 | (0.83, 1.00) | 0.06 | 5936 | 0.92 | (0.82, 1.04) | 0.19 |
| **MCS** |  | 0.83 | (0.76, 0.91) | <0.001 |  | 0.80 | (0.82, 0.89) | <0.001 |

Model 1: Age and sex. Model 5: Age, sex, socioeconomic status (manual vs non-manual labour), smoking status (current/former vs never), alcohol consumption (units), self-reported comorbidity (cancer, myocardial infarction, stroke, bronchitis, asthma, arthritis, depression), hearing problems, waist-hip ratio and systolic blood pressure. HRs represent the risk of incident dementia associated per SD change in the respective measure. N = Number of participants included in each respective model. PCS = Physical component summary, MCS = Mental component summary.

**Table S8.** Sensitivity analysis for the association between SF-36 subscale scores and incident all-cause dementia (excluding those with dementia diagnosis in first five years after 3HC).

| **Model 1** | | | | **Model 5** | | | |
| --- | --- | --- | --- | --- | --- | --- | --- |
| **SF-36 subscale** | **N** | **HR** | **95% CI** | | **N** | **HR** | **95% CI** |
| **Physical functioning** | 7028 | 0.90 | (0.75, 1.07) | | 5675 | 1.15 | (0.89, 1.49) |
|  |  |  |  | |  |  |  |
| **Role-physical functioning** |  | 0.87 | (0.72, 1.05) | |  | 0.90 | (0.71, 1.14) |
|  |  |  |  | |  |  |  |
| **Bodily pain** |  | 1.00 | (0.99, 1.01) | |  | 1.01 | (1.00, 1.02) |
| **General health** |  | 0.96 | (0.80, 1.17) | |  | 1.02 | (0.78, 1.33) |
|  |  |  |  | |  |  |  |
| **Social functioning** |  | 0.98 | (0.98, 0.99) | |  | 0.98 | (0.97, 0.99) |
|  |  |  |  | |  |  |  |
| **Role-emotional functioning** |  | 0.61 | (0.54, 0.69) | |  | 0.62 | (0.53, 0.74) |
|  |  |  |  | |  |  |  |
| **Vitality** |  | 0.98 | (0.97, 0.99) | |  | 0.98 | (0.97, 0.99) |
| **Mental health** |  | 0.64 | (0.55, 0.74) | |  | 0.59 | (0.49, 0.73) |

Model 1: Age and sex. Model 5: Age, sex, socioeconomic status (manual vs non-manual labour), smoking status (current/former vs never), alcohol consumption (units), self-reported comorbidity (cancer, myocardial infarction, stroke, bronchitis, asthma, arthritis, depression), hearing problems, waist-hip ratio and systolic blood pressure. HRs represent the risk of incident dementia associated per SD change in the respective measure. N = Number of participants included in each respective model.

**Table S9.** Association between deciles of SF-36 summary scores and incident all-cause dementia.

Model 1: Age and sex. Model 5: Age, sex, socioeconomic status, smoking status, alcohol consumption, self-reported comorbidity, hearing problems, waist-hip ratio and systolic blood pressure. Each unit change in HR represents one SD change in the respective measure. N = Number of participants included in each respective model. PCS = Physical component summary, MCS = Mental component summary.

|  | **Model 1** | | | | **Model 5** | | | |
| --- | --- | --- | --- | --- | --- | --- | --- | --- |
| **SF-36 summary measure** | **N** | **HR** | **95% CI** | **p** | **N** | **HR** | **95% CI** | **p** |
| **PCS** | 7452 |  |  |  | 5995 |  |  |  |
| **Decile 1** |  | 1.13 | (0.89, 1.43) | 0.32 |  | 1.09 | (0.79, 1.50) | 0.61 |
| **Deciles 2-9** |  | 1.00 |  |  |  | 1.00 |  |  |
| **Decile 10** |  | 0.89 | (0.60, 1.31) | 0.54 |  | 0.88 | (0.56, 1.37) | 0.58 |
|  |  |  |  |  |  |  |  |  |
| **MCS** |  |  |  |  |  |  |  |  |
| **Decile 1** |  | 1.98 | (1.55, 2.52) | <0.001 |  | 2.12 | (1.57, 2.86) | <0.001 |
| **Deciles 2-9** |  | 1.00 |  |  |  | 1.00 |  |  |
| **Decile 10** |  | 0.66 | (0.49, 0.89) | 0.007 |  | 0.65 | (0.46, 0.93) | 0.02 |

**Table S10.** Association between quintiles of SF-36 summary scores and incident all-cause dementia.

|  | **Model 1** | | | | **Model 5** | | | |
| --- | --- | --- | --- | --- | --- | --- | --- | --- |
| **SF-36 summary measure** | **N** | **HR** | **95% CI** | **p** | **N** | **HR** | **95% CI** | **p** |
| **PCS** | 7452 |  |  |  | 5995 |  |  |  |
| **Quintile 1** |  | 1.30 | (0.98, 1.71) | 0.06 |  | 1.29 | (0.93, 1.79) | 0.13 |
| **Quintile 2** |  | 1.23 | (0.93, 1.63) | 0.15 |  | 1.17 | (0.84, 1.62) | 0.35 |
| **Quintile 3** |  | 1.00 |  |  |  | 1.00 |  |  |
| **Quintile 4** |  | 1.03 | (0.75, 1.42) | 0.85 |  | 1.07 | (0.75, 1.54) | 0.70 |
| **Quintile 5** |  | 1.03 | (0.73, 1.44) | 0.86 |  | 1.03 | (0.70, 1.50) | 0.90 |
|  |  |  |  |  |  |  |  |  |
| **MCS** |  |  |  |  |  |  |  |  |
| **Quintile 1** |  | 1.51 | (1.15, 1.98) | 0.003 |  | 1.69 | (1.20, 2.36) | 0.002 |
| **Quintile 2** |  | 1.10 | (0.82, 1.46) | 0.54 |  | 1.39 | (0.99, 1.95) | 0.06 |
| **Quintile 3** |  | 1.00 |  |  |  | 1.00 |  |  |
| **Quintile 4** |  | 0.85 | (0.63, 1.16) | 0.31 |  | 1.01 | (0.71, 1.44) | 0.96 |
| **Quintile 5** |  | 0.74 | (9.55, 0.99) | 0.04 |  | 0.83 | (0.58, 1.19) | 0.32 |

Model 1: Age and sex. Model 5: Age, sex, socioeconomic status, smoking status, alcohol consumption, self-reported comorbidity, hearing problems, waist-hip ratio and systolic blood pressure. Each unit change in HR represents one SD change in the respective measure. N = Number of participants included in each respective model. PCS = Physical component summary, MCS = Mental component summary.

**Figure S1.** Crude Cumulative Incidence of dementia according to the mental component score (MCS) deciles. MCS = Mental component summary score. Decile 1 = Lowest; Decile 10 = Highest.

**
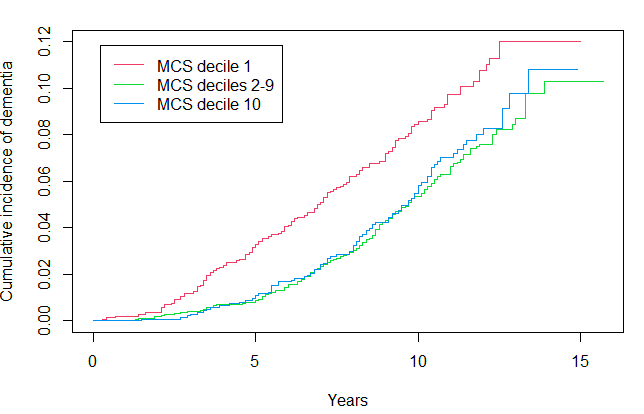
**

**
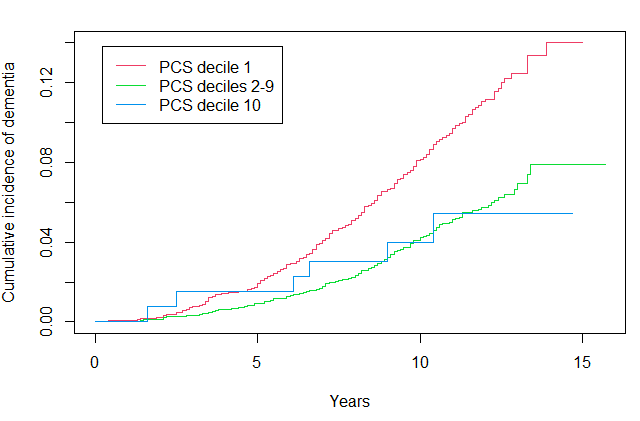
Figure S2.** Crude Cumulative Incidence of dementia according to the physical component score (PCS) deciles. PCS = Physical component summary score. Decile 1 = Lowest; Decile 10 = Highest.

**
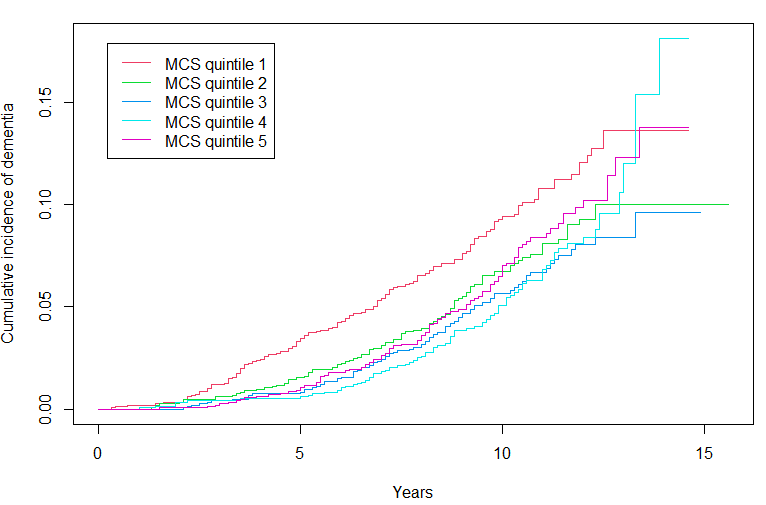
Figure S3.** Crude Cumulative Incidence of dementia according to the mental component score (MCS) quintiles. MCS = Mental component summary score. Quintile 1 = Lowest; Quintile 5 = Highest.


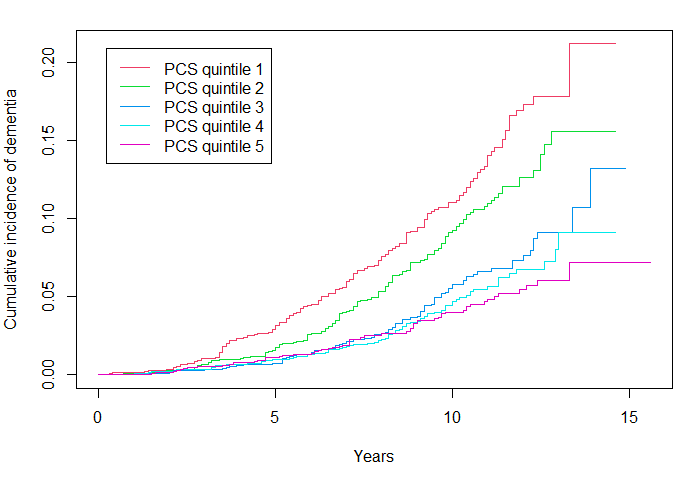
**Figure S4.** Crude Cumulative Incidence of dementia according to the physical component score (PCS) quintiles. PCS = Physical component summary score. Quintile 1 = Lowest; Quintile 5 = Highes

**Table S11.** Association between SF-36 summary scores and incident all-cause dementia, stratified by age.

|  | **Model 1** | | | **Model 2**** | | | **Model 5**** | | | | | |  |
| --- | --- | --- | --- | --- | --- | --- | --- | --- | --- | --- | --- | --- | --- |
| **Exposure** | **HR** | **95% CI** | **p** | **HR** | **95% CI** | **p** | | **HR** | **95% CI** | **p** | |  |  |
| **50-69 years** |  |  |  |  |  |  | |  |  |  |  |  |  |
| **N (n)** |  | 3946 (77) |  |  | 3946 (77) |  | |  | 3366 (61) |  | | | |
| **Average MCS*** | 0.78 | (0.65, 0.94) | 0.01 | 0.79 | (0.65, 0.95) | 0.01 | | 0.76 | (0.62, 0.94) | 0.01 | | | |
| **Average PCS*** | 0.82 | (0.68, 0.98) | 0.03 | 0.82 | (0.68, 0.99) | 0.04 | | 0.88 | (0.69, 1.12) | 0.31 | | | |
| **Change in MCS** |  |  |  |  |  |  | |  |  |  | | | |
| **Increase** | 0.81 | (0.63-1.05) | 0.11 | 0.88 | (0.47-1.65) | 0.68 | | 1.07 | (0.53-2.17) | 0.85 | | | |
| **Stable (Ref)** | 1.00 |  |  | 1.00 |  |  | | 1.00 |  |  | | | |
| **Decrease** | 1.44 | (1.14-1.82) | 0.002 | 1.66 | (0.92-2.99) | 0.09 | | 1.70 | (0.85-3.36) | 0.13 | | | |
| **Change in PCS** |  |  |  |  |  |  | |  |  |  | | | |
| **Increase** | 1.04 | (0.77-1.41) | 0.80 | 1.26 | (0.66-2.40) | 0.48 | | 1.24 | (0.59-2.60) | 0.57 | | | |
| **Stable (Ref)** | 1.00 |  |  | 1.00 |  |  | | 1.00 |  |  | | | |
| **Decrease** | 1.21 | (0.98-1.49) | 0.08 | 1.53 | (0.90-2.61) | 0.12 | | 1.38 | (0.75-2.56) | 0.30 | | |  |
| **≥70 years** |  |  |  |  |  |  | |  |  |  | | | |
| **N (n)** |  | 3266 (417) |  |  | 3266 (417) |  | |  | 2449 (295) |  | | | |
| **Average MCS*** | 0.81 | (0.75, 0.89) | <0.001 | 0.81 | (0.75, 0.89) | <0.001 | | 0.81 | (0.73, 0.89) | <0.001 | | | |
| **Average PCS*** | 0.95 | (0.86, 1.04) | 0.27 | 0.95 | (0.86, 1.04) | 0.27 | | 0.96 | (0.85, 1.10) | 0.57 | | | |
| **Change in MCS** |  |  |  |  |  |  | |  |  |  | | | |
| **Increase** | 0.82 | (0.63-1.05) | 0.11 | 0.60 | (0.44-0.82) | 0.001 | | 0.52 | (0.36-0.75) | <0.001 | | | |
| **Stable (Ref)** | 1.00 |  |  | 1.00 |  |  | | 1.00 |  |  | | | |
| **Decrease** | 1.44 | (1.14-1.82) | 0.002 | 1.44 | (1.14-1.83) | 0.002 | | 1.49 | (1.11-1.98) | 0.007 | | | |
| **Change in PCS** |  |  |  |  |  |  | |  |  |  | | | |
| **Increase** | 1.04 | (0.77-1.41) | 0.80 | 0.99 | (0.71-1.37) | 0.95 | | 0.92 | (0.62-1.35) | 0.66 | | | |
| **Stable (Ref)** | 1.00 |  |  | 1.00 |  |  | | 1.00 |  |  | | | |
| **Decrease** | 1.21 | (0.98-1.49) | 0.08 | 1.19 | (0.96-1.47) | 0.11 | | 1.04 | (0.81-1.34) | 0.77 | | | |

Model 1: Age and sex. Model 2: Age, sex, education level. Model 3: Age, sex, socioeconomic status (manual vs non-manual labour), smoking status (current/former vs never), alcohol consumption (units). Model 4: Age, sex, self-reported comorbidity (cancer, myocardial infarction, stroke, bronchitis, asthma, arthritis, depression) and hearing problems. Model 5: Age, sex, socioeconomic status, smoking status, alcohol consumption, self-reported comorbidity, hearing problems, waist-hip ratio and systolic blood pressure. N = Number of participants included in each respective model, n=number of incident cases of dementia. PCS = Physical component summary, MCS = Mental component summary. *Each unit change in HR represents one SD change in the respective measure; all co-variables measured at 3HC; **for change analyses: Models 2 and 5 are additionally adjusted for MCS/PCS scores at HLEQ1.
